# 5-HT receptor agonist Valerenic Acid enhances the innate immunity signal and suppresses glioblastoma cell growth and invasion

**DOI:** 10.7150/ijbs.44906

**Published:** 2020-05-18

**Authors:** Qingli Lu, Yuan Ding, Yang Li, Qingli Lu

**Affiliations:** 1 Department of Dermatology and Venereology, Binhaiwan Central Hospital of Dongguan, Dongguan 523000, P.R. China.; 2 Department of Dermatology and Venereology, People's Hospital of Xin jiang Uygur Autonomous Region, Urumqi 830000, P.R. China.; 3 Department of Immunology, School of Biology and Basic Medical Sciences, Soochow University, Suzhou, P.R. China.

**Keywords:** 5-HT receptor, Valerenic Acid, glioblastoma cell

## Abstract

Glioblastoma multiform (GBM) continues to threaten people's lives due to the limited therapeutic strategies. As a new drug, Valerenic Acid suppresses the progression of GBM, however, the mechanism is largely unknown. Here, we found that Valerenic Acid can inhibit cell proliferation, migration and invasion of GBM cells by increasing innate immune signals such as enhancing ROS levels and activating the AMPK pathway. Inhibition of ROS by N-acetylcysteine (NAC) or attenuation of AMPK by Compound C could block Valerenic Acid-induced cell death. Additionally, the xenograft mouse model also confirmed that Valerenic Acid had anti-tumor effect. Together, our results provide compelling rational to develop Valerenic Acid as an anti-tumor agent against GBM patients.

## Introduction

Glioblastoma multiform (GBM) is the most common and aggressive type of brain tumors in adults with the length of survival ranging from 12 to 17 months 1. Currently, the prevalent treatment is surgery followed by the combination of radiotherapy and chemotherapy 2. However, the aggressive GBM still has limited therapeutic options. Therefore, understanding the underlying mechanisms and developing promising therapeutic strategies are of necessity to battle against this disease.

Valerenic acid is one of components of Valerian plant and has been reported to alleviate the symptom of insomnia 3. It also has anti-inflammatory benefit via inhibiting the transcription of NF-κB, one essential transcription factor controlling the expression of numerous inflammation-related genes. Moreover, evidence suggests that Valerenic acid acts as an agonist for 5-HT receptors (serotonin receptors), which are G-coupled receptors that mediates neuronal activity via regulating cyclic adenosine monophosphate (cAMP) levels 3. As we known, the serotonin receptors influence various biological and neurological processes and new evidence suggest that serotonin receptors are also involved in cancer progression 5-9. However, the role of 5-HT in GBM progression and whether Valerenic acid has anti-tumor effect on GBM patients are largely unknown.

In this study, we found that one of 5-HT receptors, HTR5A, was downregulated in high grade GBM compared to low grade glioma. Valerenic acid, one potential agonist of HTR5A, had strong capacity to conduct cytotoxicity in GBM cells via suppressing cell proliferation, EMT, migration and invasion. Mechanistically, induced ROS induction and AMPK activation were responsible for Valerenic acid - induced cell death of GBM cells. The anti-tumor effect of Valerenic acid was also corroborated by in vivo xenograft GBM mouse model. Taken together, Valerenic acid may serve as a potential anti-tumor agent towards GBM patients.

## Materials and methods

### Cell lines and reagent

LN229 (CRL-2611), U251 MG human glioblastoma cell lines and MDA-MB-231 were purchased from ATCC. Cells were cultured in Dulbecco's modified Eagle's medium (DMEM) (Corning, 10-013-CV) supplemented with 10% Fetal Bovine Serum (Gibco, 10437028) and 1% Antibiotic-Antimycotic Solution (Corning, 30-004-CI). NHA (CC-2565) were from LONZA. NHA were cultured in NHA growth medium according to the standard protocol as described in the instructions for the Human Astrocyte Cell System (LONZA). Valerenic acid were purchased from Selleckchem Company. The CC3, p-AMPK alpha, p-AMPK beta, p-ACC, p-AKT (473) antibodies were purchased from Cell Signaling Technology (Beverly, Massachusetts, USA).

### Mice

1x10^6^ cells luciferase- LN299 and -U251 MG cells were intracranially injected into six- to eight-week old female nude mice (Nu(NCr)-Foxn1nu, from Charles River, Strain Code: 490). Tumor size was measured by digital caliper twice per week. Five mice per group were used. All of our animal experiments were approved by Xinjiang Hospital Ethical Committee, the ethics approval number is KY2018118170.

### Cell viability assay

Cell viability was measured using 3-(4, 5-dimethylthiazolyl)-2 and 5-diphenyltetrazolium bromide (MTT) assay. LN229 and U251 MG cells were seeded in 96-well plates at a density of 5000 cells per well. 24 h later, cells were treated with Valerenic acid at a 2% FBS containing medium for another 24 h. Then, 25 μl of MTT (Sigma, St Louis, Missouri, USA) was added to each well and incubated for 4 h. The solution was removed and 50 μl dimethyl sulfoxide was added to each sample. We measured the absorbance at 570 nm to calculate cell viability after incubation at 37°C for 10 min. Cells were pre-treated with either N-Acetyl-L-cysteine (A7250, Sigma) or Compound C (171260, CAS 866405-64-3, Calbiochem) prior to Valerenic acid lerenic acid treatment.

### Intracellular reactive oxygen species (ROS) assay

The intracellular ROS level was determined by using dichlorofluorescein-diacetate (DCFH-DA) fluorescent probe. LN229 and U251 MG cells were treated with various concentration of Valerenic acid for 30min. After treatment, cells were darkly incubated with DCFH-DA (10 μM) in fresh culture medium at 37 °C for 30 min. Then fluorescence intensities were recorded. H2O2 was used as positive control.

### Wound-healing assay

Migration of GBM cells was detected using the wound-healing assay. LN229 and U251 MG Cells were seeded in 6 cm dishes and incubated overnight. When cells grew into a confluent monolayer in the plate, we scraped the cells by a sterile micropipette tip. The cells were washed twice and the cells were incubated with or without Valerenic acid for 24 h, 48h. Images were captured using a microscopic camera system (Carl Zeiss, Oberkochen,o Baden-Württemberg, Germany).

### 3D spheroid invasion assays

LN229 and U251MG cells were trypsinized, resuspended and counted. 50 cells total were seeded in neural sphere medium (DMEM/F12, Corning #15-090-CV, Lglutamine, 2mM, Invitrogen #25030-081, N-2 supplement, 1X, Invitrogen #17502048, B-27 Supplement, 1X, Invitrogen #17504044, BSA, 50ug/ml, Sigma, EGF & bFGF, 20 ng/ml each, R&D systems, Antibiotic-Antimycotic Solution, 1% Corning #30-004-CI) per well in 96-well Ultra Low Cluster Plates (Costar). After 24-48 hours, medium was replaced with regular culture medium containing 10% FBS. Two days after plating spheroids were harvested and embedded into Basement Membrane Matrix (Corning #356234). Invasion was monitored and quantified by measuring the area occupied by cells using ImageJ software.

### Cell apoptosis assay

Cell apoptosis was detected using the Annexin V-FITC Apoptosis Detection Kit (KeyGEN). LN229 and U251 MG cells were seeded in 6 cm dishes (5 × 10^4^ cells per well) and treated with various concentration of Valerenic acid for 24 h. Then cells were harvested, washed with cold PBS, and centrifuged. Cells were incubated with Propidium iodide/Annexin for 10 min and subjected to flow cytometry analysis.

### Western blotting

Cells were lysed by PIPA lysis buffer. Equal amount of protein was subjected to 8%-12% SDS-PAGE gel separation and transferred to a polyvinylidene difluoride membrane. After being blocked by 5% skim milk, the membranes were incubated with a specific primary antibody at 4°C overnight. Then the membranes were washed by Tris-buffered saline with 0.1% Tween-20 and incubated with the secondary antibody at room temperature for 2 h. Blots were detected using the ChemiDOC XRS + system (Bio-Rad Laboratories, Hercules, California, USA).

### Statistical analysis

Statistical analysis was carried out using GraphPad Prism 6.0 software (GraphPad Software, San Diego, California, USA). Data were presented as mean ± SE. Differences were analyzed using the Student t-test, and significance was set at P value less than 0.05. Each experiment was conducted independently, and repeated at least three times.

## Results

### HTR5A was downregulated in high grade Glioma

**Figure 1:**
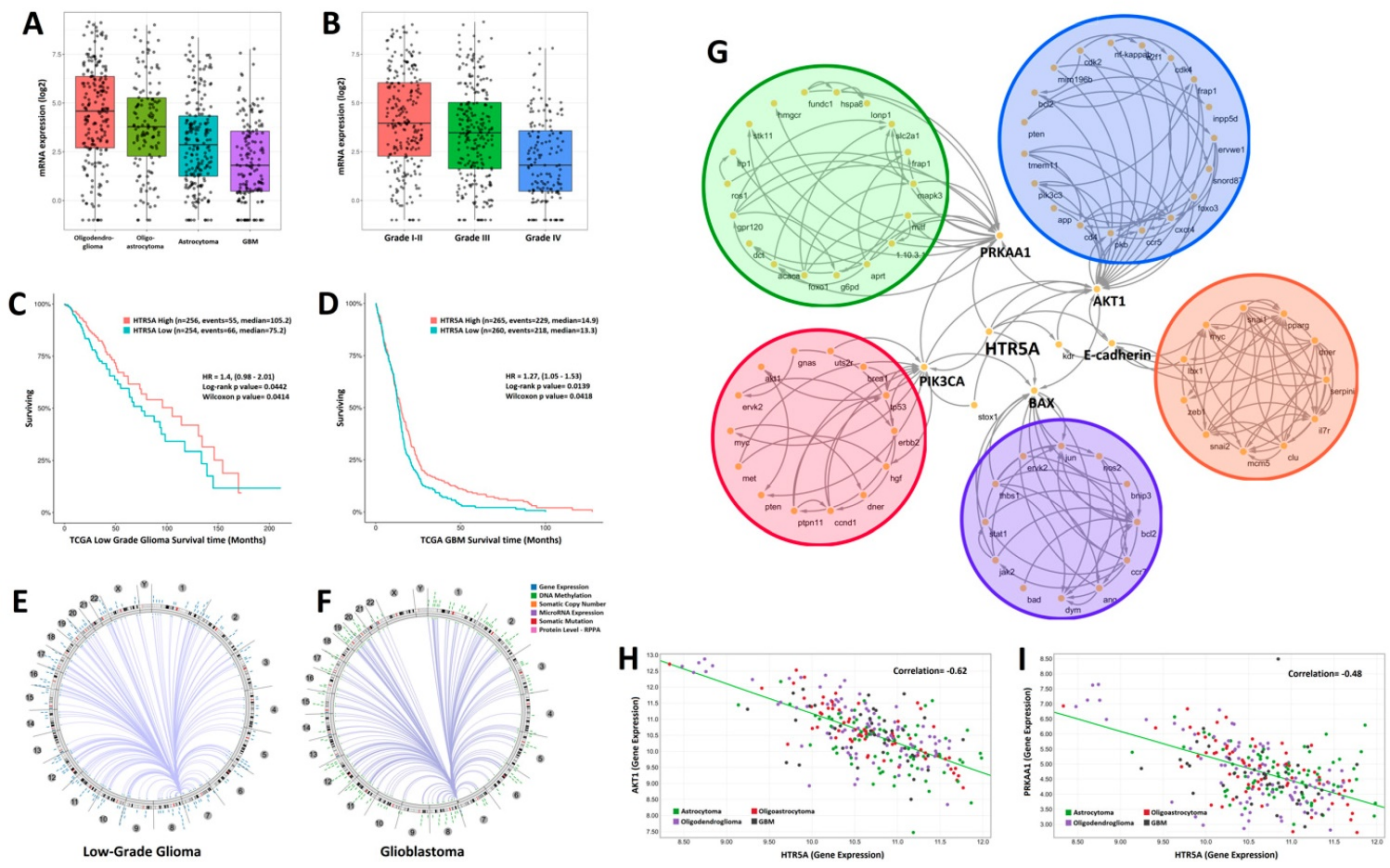
HTR5A was downregulated in GBM patients compared to other pathological subtypes of glioma. A. The mRNA levels of HTR5A were decreased in GBM patients compared to other subtypes of glioma based on TCGA datasets. B. TCGA datasets also showed that HTR5A is decreased in Grade IV glioma. C-D. Kaplan-Meier survival curve analysis of the TCGA datasets indicated that HTR5A is a negatively prognostic factor for overall survival in glioma patients. E-F. Circos analysis of epidermal changes in HTR5A-associated genes in WHO I-III gliomas and GBM. G. KEGG analysis revealed that the HTR5A protein was involved in five signaling transduction pathways including the AMPK. H. Pearson Correlation Coefficient Analysis of HTR5A and PRKAA1.

By analyzing the genome-wide sequencing data of human glioma samples provided by TCGA, we found that the expression levels of HTR5A was decreased in glioblastoma patients compared to low-grade gliomas including oligodendroglioma, oligoastrocytoma, and astrocytoma (Fig. 1A). Further analysis suggested that HTR5A had a lowest level in WHO grade IV glioma compared to others grade glioma (Fig. 1B). TCGA dataset also revealed that HTR5A could serve as a good prognostic factor for overall survival (Fig. 1C-D). In addition, we used Circos to show the density distribution of the various region change loci associated with HTR5A in the genome. As shown in Fig.1E and Fig.1F, the results correlated with a variety of data, including gene expression, sRNA, TE (transposable element), and methylation density distribution. We found significantly different patterns of structural variation in HTR5A between WHO I-III glioma and GBM. To explore which signaling pathways are related to HTR5A, we performed Kyoto Encyclopedia of Genes and Genomes (KEGG) pathway analysis and the results showed that HTR5A was involved in six main pathways (AKT, PRKAA1, PIK3CA, EMT and BAX). The metabolic pathway of these genes was analyzed and the genes were labeled with different colors (Fig. 1G). Among these pathways, PRKAA1, also called AMPK, was received our attention. By processing Pearson correlation analysis to calculate the expression of HTR5A and PRKAA1 in different WHO grading gliomas, we found that the expression levels of HTR5A had a linear correlation with the PRKAA1 in glioma specimens, r=0.48, P <0.05 (Fig. 1H).

### Valerenic Acid induces cytotoxicity via apoptosis/autophagy in Glioblastoma cells

**Figure 2:**
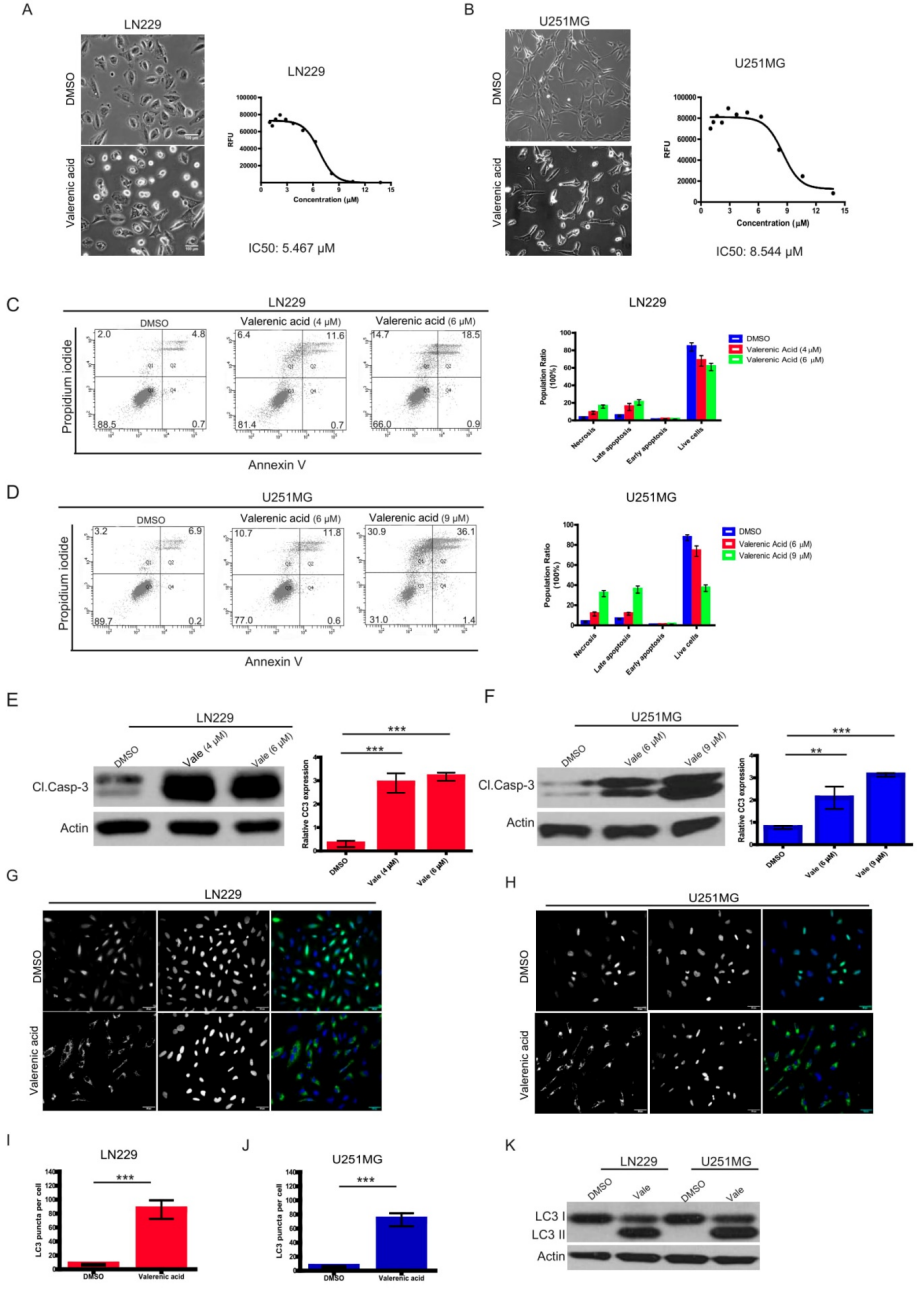
Valerenic Acid inhibited cell survival of GBM cells. A-B. LN229 (A) and U251 MG (B) cells were treated with or without Valerenic Acid for 24h. Left, representative images from three independent experiments were shown. Right, IC50 of Valerenic Acid was provided. Scale bar =100 μm. C-D. LN229 (C) and U251 MG (D) cells with or without Valerenic Acid treatment were subjected to flow cytometry analysis. E-F. Cleaved caspase 3 (CC3) was measured by western blotting analysis after cells were treated with VA for 24h. G-H. Immunofluorescent staining of GFP-LC3 was performed in LN229 (G) and U251 MG (H) cells with or with VA treatment. I-J. Quantification analysis of GFP-LC3 puncta in LN229 (I) and U251 MG (J) cells before and after VA treatment. K. The protein levels of LC3-II were examined in these two cell lines. This experiment was done by three times and statistical analyses were made. *P<0.05, **P<0.01, ***P<0.001

Given the fact that HTR5A was downregulated in GBM patients, we sought to explore whether its agonist, Valerenic Acid (Valerenic acid in short), has capacity to prevent GBM progression. To end this, we treated glioblastoma cell lines, LN229 and U251 MG, with Valerenic acid in short for 24 hours. Results showed that Valerenic acid significantly inhibited the cell viability of LN229 and U251 MG, with IC50 value of 5.467 ± 0.07 μM and 8.544 ± 0.72 μM, respectively (**Fig. 2A-B**). Interestingly, we also noticed that cell morphology of LN229 and U251 MG was altered upon Valerenic acid treatment: small population of cells was detached from culture dish and the remaining cells became rounded up, which were hallmarks of cell apoptosis. To confirm this, we performed flow cytometry analysis on GBM cells before and after Valerenic acid treatment by using standard Annexin V/PI staining. Results revealed that much more apoptotic cells were observed in LN229 and U251 MG cells in the presence of Valerenic acid when compared with the untreated cells (**Fig. 2C-D**). Also, Valerenic acid-induced cell apoptosis was corroborated by protein detection of cleaved caspase 3 (CC3) (**Fig. 2E, F**), which showed that Valerenic acid dramatically boosted CC3 expression in both cell lines. In addition, we found that autophagy was also involved in Valerenic acid-mediated cell death, which was validated by increased number of LC3 puncta (**Fig. 2G-J**) and elevated LC3-II/LC3-I ratio (**Fig. 2K**) upon Valerenic acid treatment. We also examined the effect of Valerenic acid in normal human astrocytes (NHA) and breast cancer cell line MDA-MB-231 and found that it can also inhibit their survival similarly as LN229 and U251MG (Figure S1A,B). Taken together, these results indicate that Valerenic acid mediated GBM cell death via activating apoptosis and autophagy pathways.

### Valerenic Acid triggers oxidative stress in Glioblastoma cells

**Figure 3:**
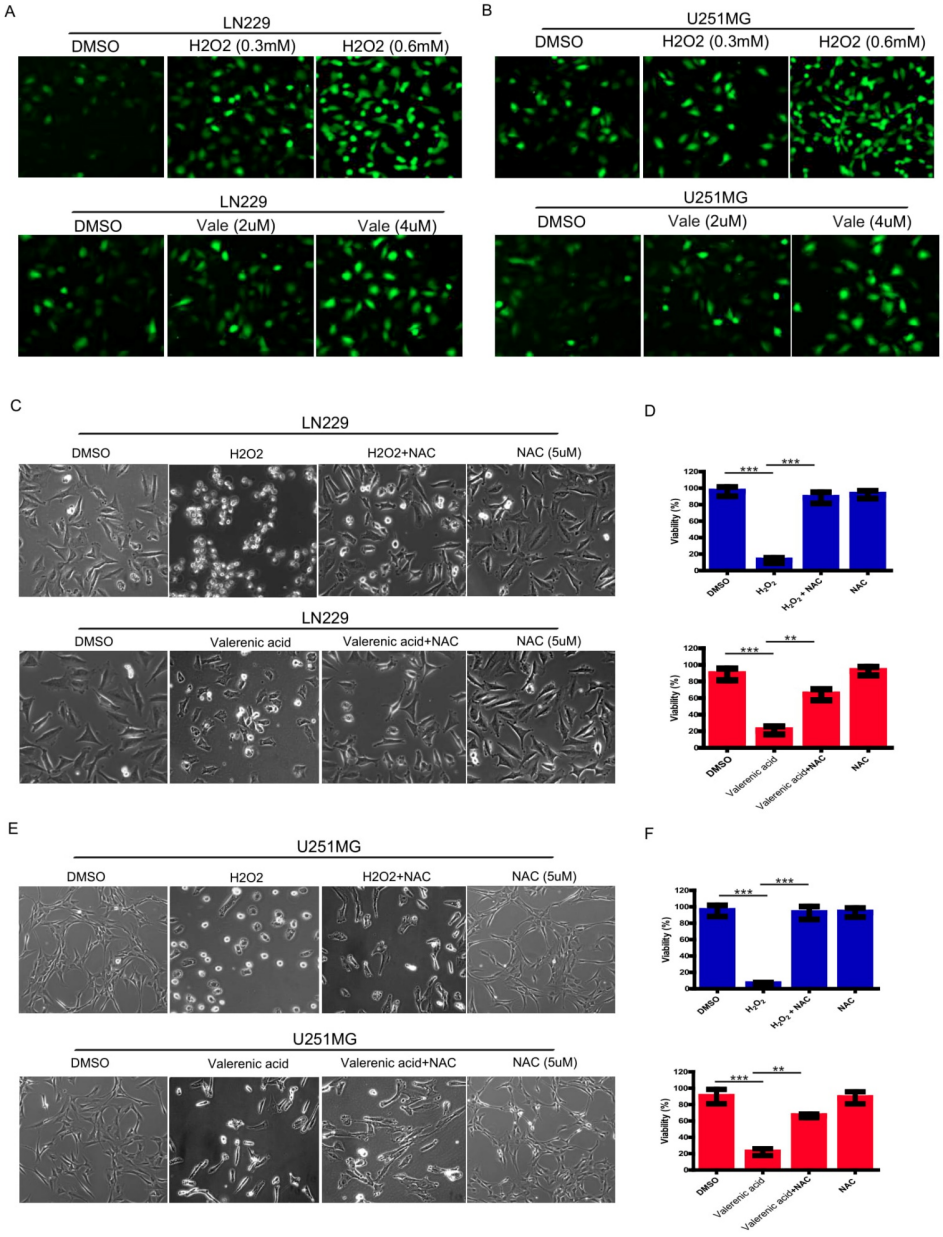
Valerenic Acid triggered oxidative stress GBM cells. A-B. Cells were treated with H2O2 (0, 0.3, 0.6 mM) or Valerenic Acid (0, 2, 4 μM) for 30min and then incubated with DCFH-DA fluorescent probe for 30 min. Representative images from three independent experiments were provided. C-D. NAC (5 μM) could reverse H2O2 and Valerenic Acid induced ROS levels in LN229 cells. Left, representative images. Right, statistical analysis. E-F. NAC (5 μM) could reverse H2O2 and Valerenic Acid induced ROS levels in U251 MG cells. Left, representative images. Right, statistical analysis. *P<0.05, **P<0.01, ***P<0.001.

Reactive oxygen species (ROS) serves as a good indicator of apoptosis under both physiologic and pathologic conditions 10. To examine whether ROS was involved in Valerenic acid-induced apoptosis, intracellular ROS levels were measured by CM-H2DCFDA incorporation. As shown in **Fig. 3A, B**, GBM cells treated with Valerenic acid (2 µM, 4 µM) for 30min had an elevated ROS levels compared to untreated cells. As a positive control, H2O2 (0.3 mM, 0.6 mM) dramatically induced ROS levels in both LN229 and U251 MG cells. NAC (*N*-acetyl-L-cysteine), a synthetic precursor of intracellular cysteine and glutathione, is a well-known inhibitor of ROS due to its free radical scavenging property 11. To confirm whether Valerenic acid-mediated apoptosis was partially caused by increased intracellular ROS levels, we pre-treated LN229 and U251 MG cells with 5 µM NAC for 1 h prior to Valerenic acid treatment. Results from **Fig. 3C-F** demonstrated that NAC significantly attenuated Valerenic acid-induced cell death in LN229 cells. Similar results were obtained in U251 MG cells. As positive control, H2O2-induced cell death was totally blocked by NAC in both cell lines. These results support the notion that Valerenic acid triggers ROS elevation to promote cell death.

### AMPK activation is involved in Valerenic Acid induced cell death

**Figure 4:**
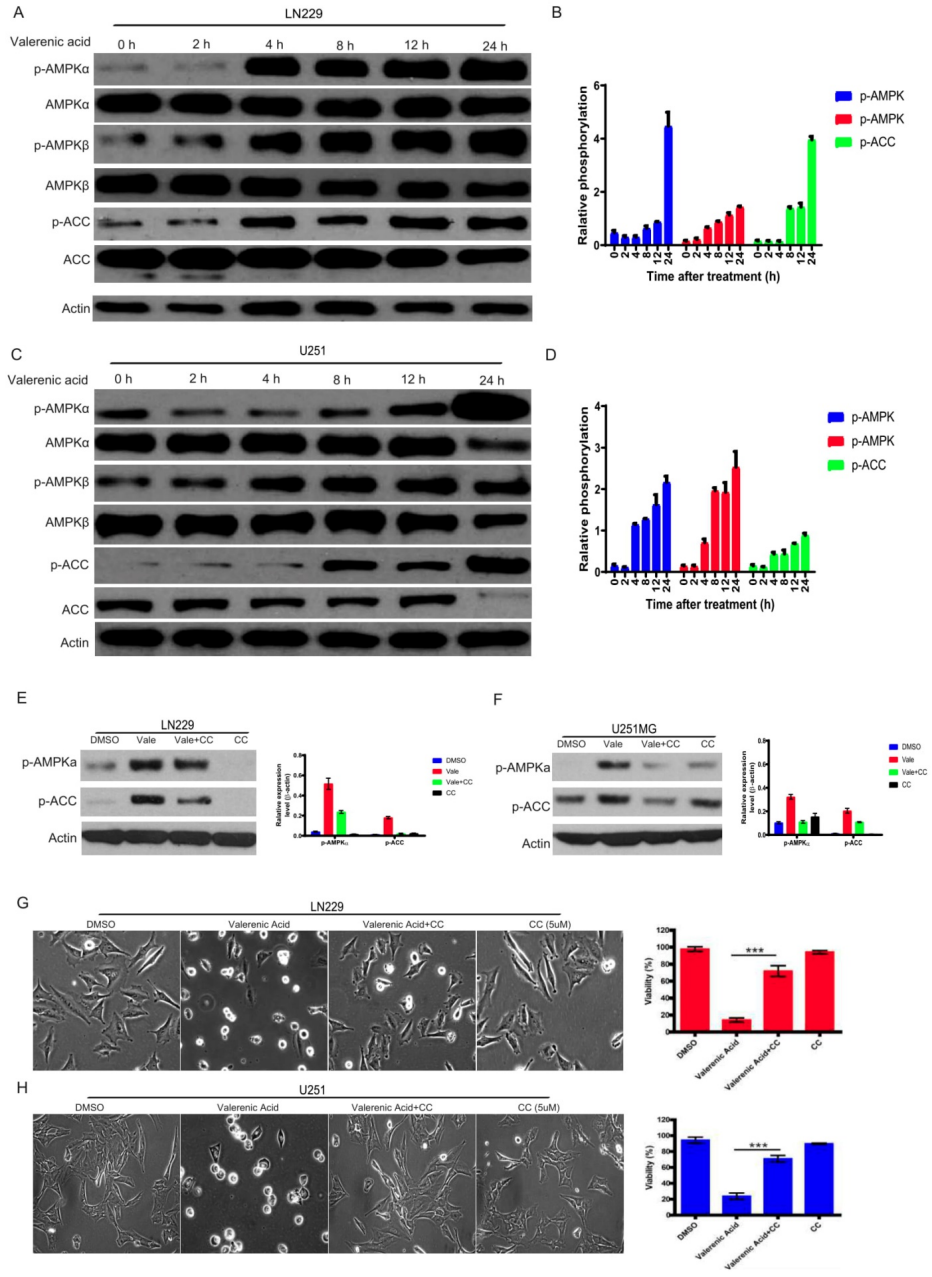
AMPK pathway was involved in Valerenic Acid mediated cell death in GBM cells. A-B. Western blot showed that Valerenic Acid could increase the phosphorylation levels of AMPK alpha, AMPK beta and ACC in LN229 cells. Actin was served as loading control. C-D. Western blot result demonstrated that Valerenic Acid elevated the phosphorylation levels of AMPK alpha, AMPK beta and ACC in U251 MG cells. Actin was served as loading control. E-F. The activation of AMPK pathway by Valerenic Acid treatment was blocked in the presence of Compound C. G-H. Valerenic Acid-induced cell death was partially reversed by supplementing LN229 (G) or with U251 MG (H) cell with Compound C. Representative images from three independent experiments were provided. *P<0.05, **P<0.01, ***P<0.001

The activity of AMP-activated protein kinase (AMPK) mirrors intracellular energy status and HTR5A was involved in AMPK pathway based on TCGA datasets. We sought to examine whether AMPK pathway was required for Valerenic acid-induced cell death. To this end, we evaluated the phosphorylation levels of AMPK alpha, AMPK beta and ACC before and after Valerenic acid treatment. As shown in **Fig. 4A-D**, Valerenic acid evidently boosted the phosphorylation levels of these three kinases in a time-dependent manner, implying activation of AMPK pathway may be involved Valerenic acid-induced cell death. To confirm this, we pre-treated GBM cells with AMPK inhibitor, Compound C (CC), and test the contribution of Valerenic acid to cell death. Expectedly, Valerenic acid-induced cell death, in a company of AMPK activation, was partially reversed in the presence of Compound C (**Fig. 4E-H**). Surprisingly, Valerenic acid has no any effects in AMPK signal in both NHA and MDA-MB-231 cell lines, although it inhibits their survival (Figure S1C). This suggests Valerenic acid displays a cell-type dependent role in AMPK activation. Collectively, these data suggest activation of AMPK pathway is one of mechanisms responsible for Valerenic acid-induced cell death.

### Valerenic Acid suppresses cell proliferation and Epithelial-Mesenchymal Transition (EMT) of GBM cells

**Figure 5:**
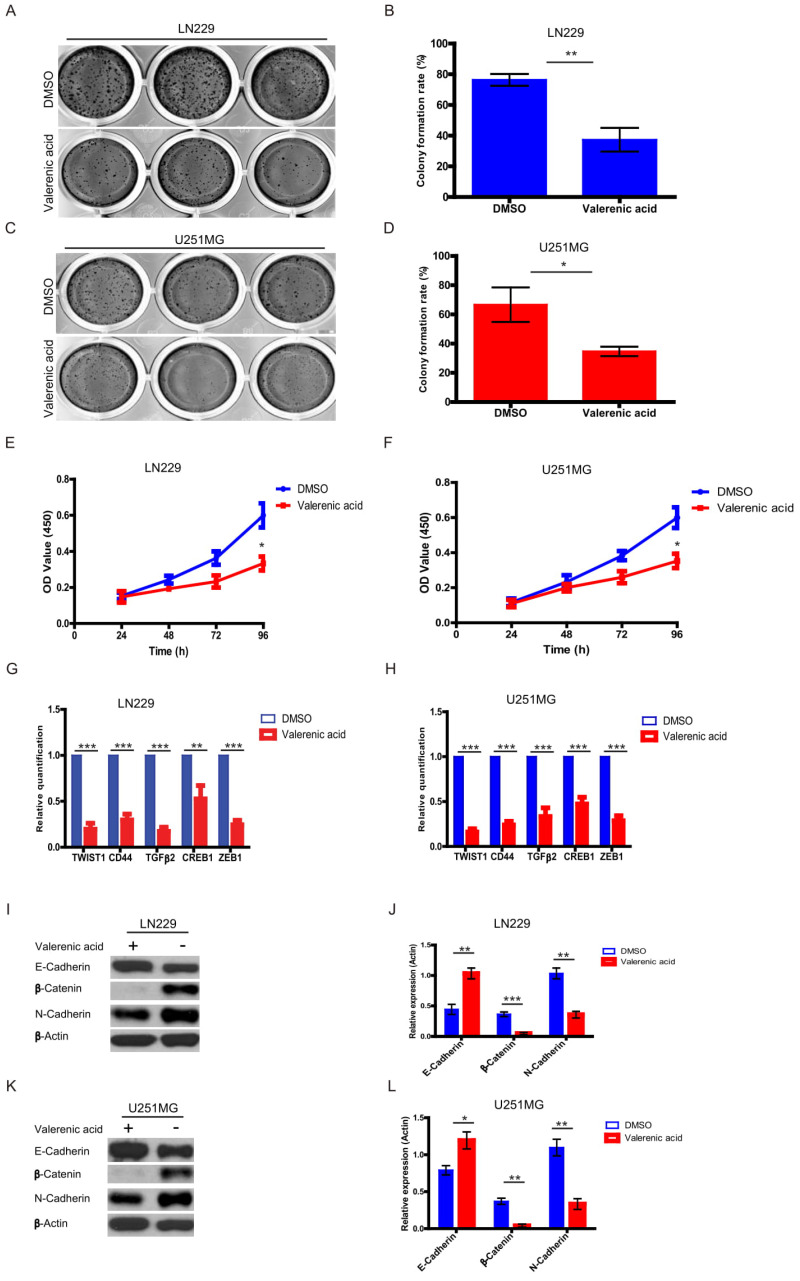
Valerenic Acid inhibited cell proliferation and Epithelial-Mesenchymal Transition (EMT). A-B. Colony formation assay revealed that Valerenic Acid significantly suppressed cell proliferation of LN229 (A, B) and U251 MG (C, D) cells. Left, representative images of colonies. Right, statistical analysis of Valerenic Acid-affected colony number. E-F. MTT results showed that Valerenic Acid suppressed cell proliferation of LN229 (E) and U251 MG (F) cells. G-H. mRNA levels of CD44, TGFbeta, TWIST, CREB and ZEB1 in both LN229 (G) and U251 MG (H) cells were evidently reduced in the presence of Valerenic Acid. Gene expression was normalized to GAPDH. I-L. Western blot results demonstrated that Valerenic Acid decreased EMT of LN229 (I, J) and U251 MG (K, L) cells, monitored by expression levels of E-cadherin, N-cadherin and beta-catenin. Actin served as loading control. Statistical analyses were also conducted. *P<0.05, **P<0.01, ***P<0.001

Higher proliferating rate and EMT induction are two important hallmarks of cancer cells. Here, by performing colony formation assay (**Fig. 5A-D**) and MTT assay (**Fig. 5E-F**), we confirmed that Valerenic acid had strong capacity to reduce cell proliferation in both LN229 and U251 MG cells. Meanwhile, several EMT-related markers were detected by either qPCR or western blot before and after Valerenic acid treatment.

Results from (**Fig. 5G-H**) suggested that Valerenic acid evidently downregulated the expression levels of CD44, TGFbeta, TWIST, CREB and ZEB1 in both LN229 and U251 MG cells. Consistently, Valerenic acid decreased the expression levels of N-cadherin, β-Catenin while had an opposite effect on the expression levels of E-cadherin, monitored by western blotting analysis (**Fig. 5I-L**). Moreover, Valerenic acid treatment also inhibits MDA-MB-231 colony formation and migration (Figure S1D, E). Together, Valerenic acid reduces GBM cell proliferation and suppresses EMT.

### Valerenic Acid inhibits cell migration and invasion of GBM cells

**Figure 6:**
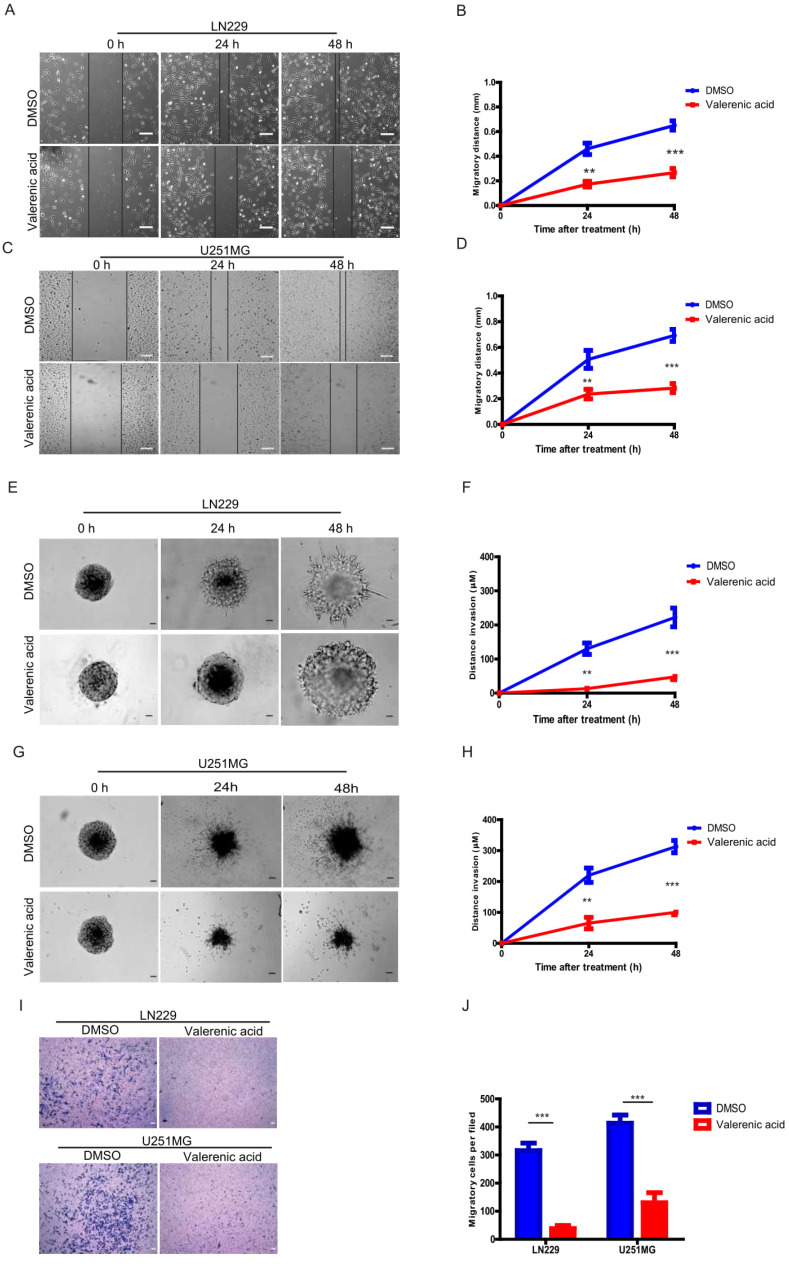
Valerenic Acid suppressed cell migration and invasion of GBM cells. A-B. The wound healing assay was conducted in LN299 cells after Valerenic Acid treatment. A, representative images from three independent experiments. B, Statistical analysis of migrating cells. C-D. The wound healing assay was conducted in U251 MG cells after Valerenic Acid treatment. C, representative images from three independent experiments. D, Statistical analysis of migrating cells. E-F. 3-D invasion assay showed that Valerenic Acid reduced cell invasion of LN299 cells. E, representative images from three independent experiments. F, Statistical analysis of invading cells. G-H. 3-D invasion assay showed that Valerenic Acid reduced cell invasion of U251 MG cells. G, representative images from three independent experiments. H, Statistical analysis of invading cells. I-J. Transwell invasion assay confirmed that Valerenic Acid suppressed cell invasion of LN299 and U251 MG cells. I, representative images from three independent experiments. J, Statistical analysis of invading cells. Scale bar=100μm. *P<0.05, **P<0.01, ***P<0.001

Given the fact that Valerenic acid suppresses the EMT of GBM cells, we sought to explore whether it affected cell migration and invasion of GBM cells. Expectedly, result from wound-healing assay demonstrated that Valerenic acid remarkably inhibited the cell migrating ability of LN229 and U251MG cells as shown in (**Fig. 6A-D**)**.** In addition, Valerenic acid suppressed cell invasion of these two cell lines based on 3-D invasion assay, which showed that more acini-like structures were observed in Valerenic acid-treated LN229 and U251 MG cells (**Fig. 6E-H**). Similar results were also obtained when we applied the Boyden chamber invasion assay to detect the invasive ability of GBM cells before and after Valerenic acid treatment (**Fig. 6I-J**). Taken together, these results indicate that Valerenic acid bears the capacity to inhibit the migrating and invasive ability of GBM cells.

### Valerenic Acid suppresses xenograft GBM tumor

**Figure 7:**
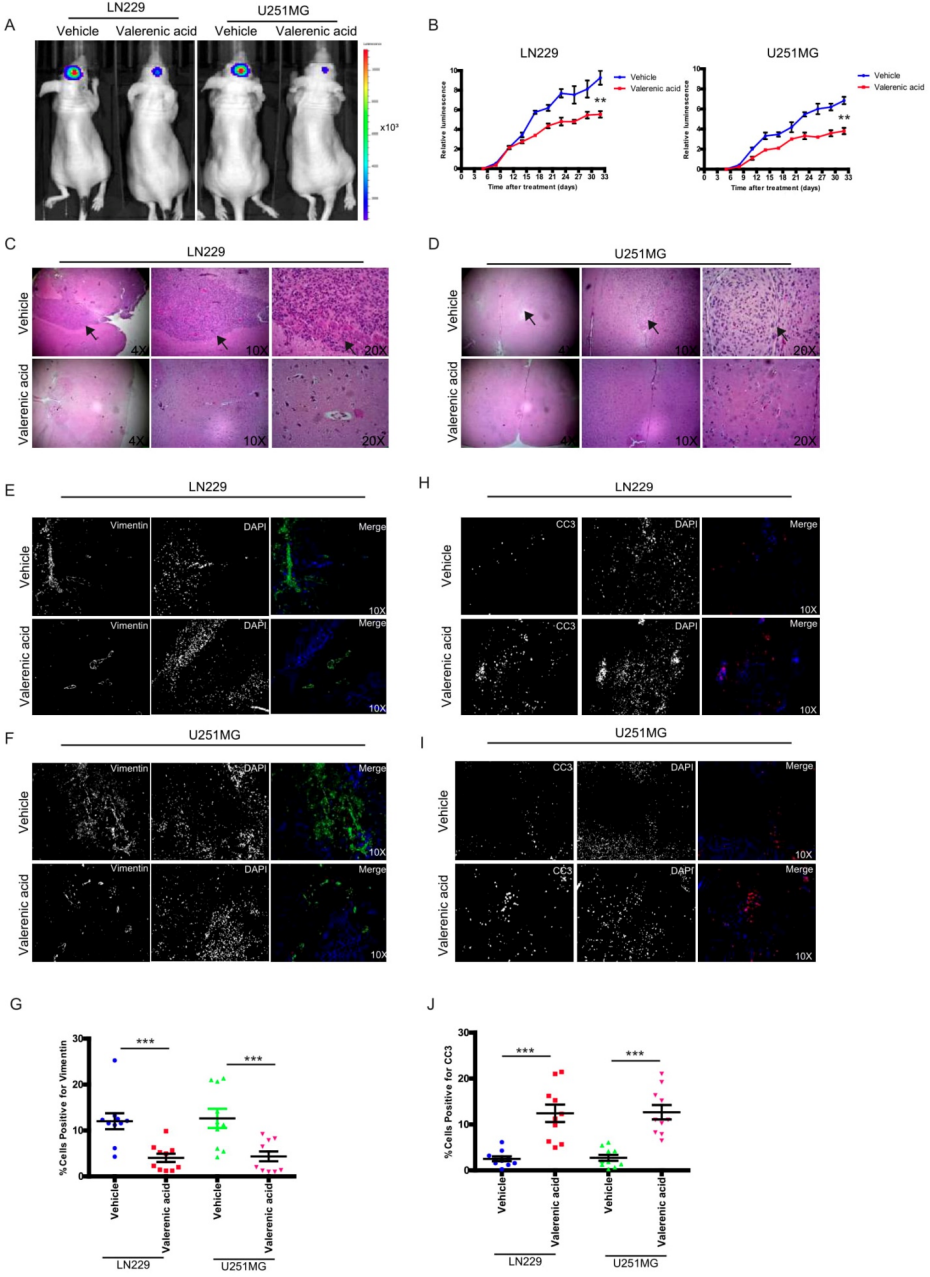
Valerenic Acid suppressed GBM tumor growth. A-B. Valerenic Acid remarkably suppressed LN299 and U251MG xenograft tumors. A, representative IVIS images. B, Statistical analysis of luciferase signal. C-D, HE staining revealed that small tumor size was observed in Statistical analysis of migrating cells-treated tumors. E-F. Immunohistochemical staining of vimentin in frozen sections derived from vehicle or Valerenic Acid-treated tumors. H-I. Immunohistochemical staining of CC3 in frozen sections derived from vehicle or Valerenic Acid-treated tumors. G, J. Statistical analyses of vimentin (G) and CC3 (J) staining. *P<0.05, **P<0.01, ***P<0.001

To pre-clinically translate our in vitro findings, we subcutaneously implanted luciferase-LN229 and luciferase-U251 MG cells into nude mice. After tumors formed, we I.P. administrated Valerenic acid or vehicle every other day (n=5 for each group). Tumors were monitored either by IVIS twice per week. As shown in (**Fig. 7A-B**), Valerenic acid treatment significantly suppressed tumor growth of LN229 and U251 MG xenograft model. HE staining also confirmed that tumor size became much smaller after Valerenic acid administration (**Fig. 7C-D**). Of note, the body weight of Valerenic acid-treated mice was indiscriminate to that of vehicle-treated ones (data not shown). To confirm molecular alterations as we observed in cell lines, we sought to detect expression levels of vimentin and CC3 by immunochemistry staining. **Fig. 7E-G** showed that less positive cells stained with vimentin were observed in frozen sections derived from Valerenic acid-treated mice, suggesting Valerenic acid indeed suppressed EMT process in vivo. Moreover, more CC3 positive cells were noticed in frozen sections derived from Valerenic acid-treated mice (**Fig. 7H-J**), indicating Valerenic acid enhanced apoptosis in xenograft GBM mouse model. Together, all these results indicate that Valerenic acid displays protective role against glioblastoma tumor growth in vivo.

## Discussion

Glioblastoma multiform (GBM) is the most common and deadliest of malignant primary brain tumors in adults. It frequently occurs at an average age of 64 12 with incidence at 3.2/100 000 13. GBMs are usually classified as primary or secondary malignant brain tumor. Most of GBMs are primary tumor and occur in aged people. Primary GBMs display much poorer prognosis than secondary GBMs 14. Although traditional therapeutic strategy such as surgery followed by radiation and chemotherapy has been improved, GBM patients still have a poor prognosis with a median survival of 15 months and the 5-year overall survival (OS) of GBM is only 9.8% 15, 16. Therefore, identifying new therapeutic targets and developing novel therapeutic strategy are of necessity to improve GBM treatment.

**Figure 8:**
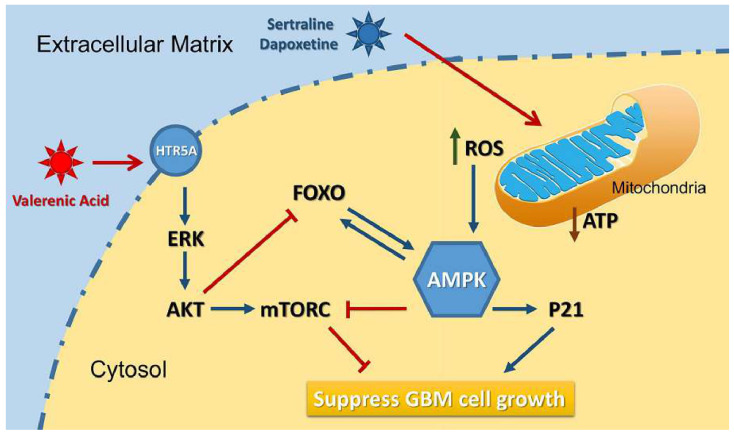
A diagram of the mechanism indicating that Valerenic Acid is an essential agonist of 5-HT receptor that can suppress glioma invasion and proliferation via elevated ROS levels and AMPK activation.

Based on the data from Cancer Genome Atlas (TCGA) database, we observed a downregulation of HTR5A in Glioblastoma patients, suggesting it may play negative role in GBM development. Indeed, HTR5A agonist, Valerenic acid, displayed suppressive effects on GBM progression both in vitro and in vivo. Mechanistically, as shown in Figure 8, Valerenic acid-induced cell death was caused by enhanced oxidative stress and energy stress pathway. Notably, antioxidant NAC and AMPK inhibitor Compound C could efficiently reverse Valerenic acid induced cell death. It is usually accepted that AMPK plays a suppressive role in cancer. However, recent study reported that cancer-associated stress chronically activated AMPK pathway in GBM stem cells, which promoted tumor growth 17. Another document also reported that AMPK was constitutively activated in astrocytes expressing oncogenic H-Ras^V12^ 18. These inconsistent results may be explained by their targeted cells different from ours or others. Functionally, Valerenic acid suppressed cell migration and invasion in vitro and effectively inhibited the tumor growth in vivo. Overall, our findings not only identify Valerenic acid as an anti-tumor agent towards GBM but also provide the mechanism by which ROS and AMPK pathway participated into Valerenic acid-induced cell death.

In summary, our data strongly suggest that Valerenic acid could suppress the survival of GBM in vitro and attenuate tumor growth in vivo. This provides compelling rational to apply Valerenic acid as anti-tumor agent to treat GBM patients.

## Supplementary Material

Supplementary figures and tables.
