# Supplementary material for: 5-HT receptor agonist Valerenic Acid enhances the innate immunity signal and suppresses glioblastoma cell growth and invasion
Source: Int J Biol Sci. 2020 May 18;16(12):2104–15. doi: 10.7150/ijbs.44906 (PMC7294948; doi:10.7150/ijbs.44906)
Supplement: Supplementary file 1 — Supplementary figures and tables. [file ijbsv16p2104s1.pdf]

Figure S1 Valerenic Acid inhibited both NHA and MDA-MB-231 cell proliferation and migration. A-B. Cells were treated with or without Valerenic Acid for 24h. IC50 of Valerenic Acid was provided. C. Western blot showed that Valerenic Acid has no effect in the phosphorylation levels of AMPK alpha, and ACC in MDA-MB-231 cells. Actin was served as loading control. D. Colony formation assay revealed that Valerenic Acid significantly suppressed cell proliferation of MDA-MB-231. E. Transwell invasion assay showed that Valerenic Acid suppressed cell invasion of MDA-MB-231.

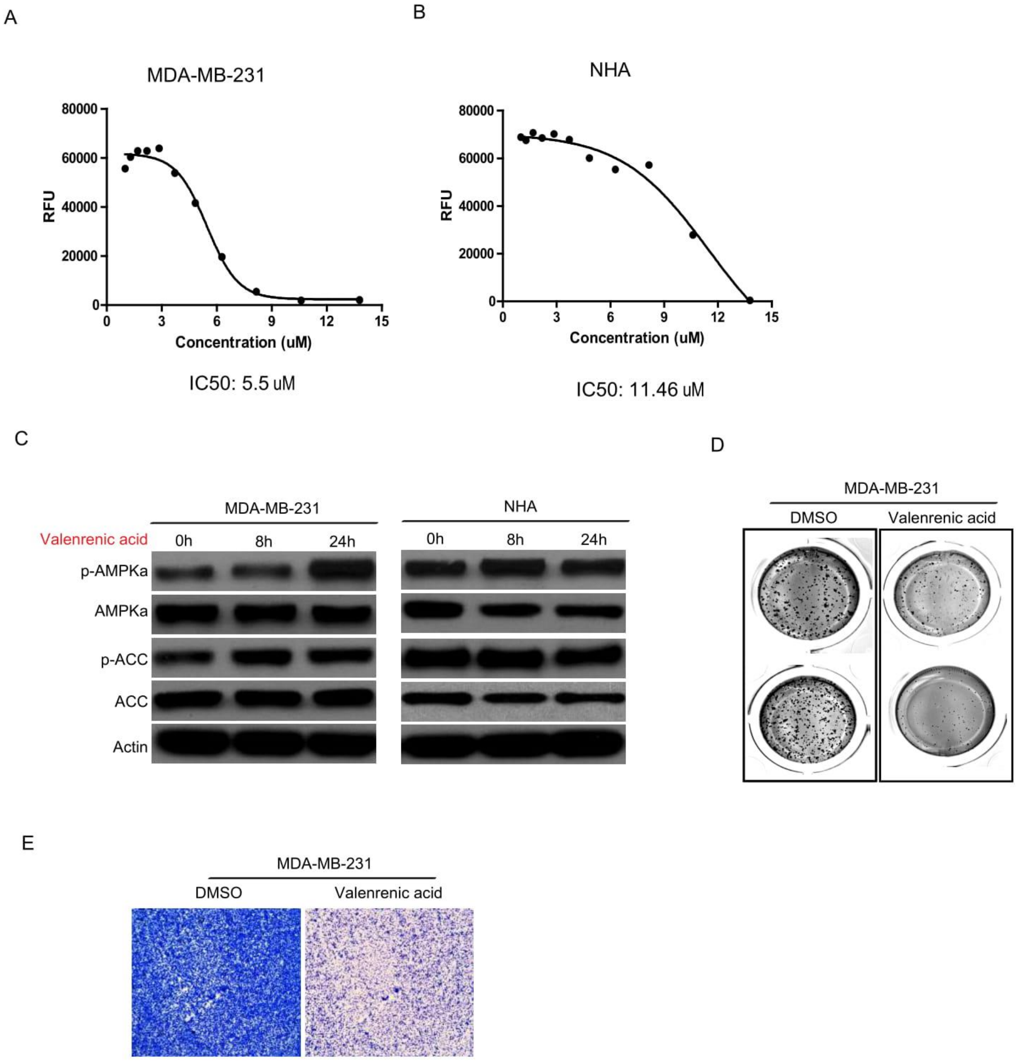
A
MDA-MB-231
RFU
80000
60000
40000
20000
0
0 3 6 9 12 15
Concentration (uM)
IC50: 5.5 uM
B
NHA
RFU
80000
60000
40000
20000
0
0 3 6 9 12 15
Concentration (uM)
IC50: 11.46 uM
C
MDA-MB-231
NHA
Valenrenic acid
0h 8h 24h
0h 8h 24h
p-AMPKa
AMPKa
p-ACC
ACC
Actin
D
MDA-MB-231
DMSO
Valenrenic acid
E
MDA-MB-231
DMSO
Valenrenic acid
